# Airway bacteria drive a progressive COPD-like phenotype in mice with polymeric immunoglobulin receptor deficiency

**DOI:** 10.1038/ncomms11240

**Published:** 2016-04-05

**Authors:** Bradley W. Richmond, Robert M. Brucker, Wei Han, Rui-Hong Du, Yongqin Zhang, Dong-Sheng Cheng, Linda Gleaves, Rasul Abdolrasulnia, Dina Polosukhina, Peter E. Clark, Seth R. Bordenstein, Timothy S. Blackwell, Vasiliy V. Polosukhin

**Affiliations:** 1 Division of Allergy, Pulmonary and Critical Care Medicine, Department of Medicine, Vanderbilt University School of Medicine, T-1218 MCN, Nashville, Tennessee 37232-2650, USA; 2 Department of Cell and Developmental Biology, Vanderbilt University School of Medicine, T-1218 MCN, Nashville, Tennessee 37232-2650, USA; 3 Rowland Institute, Cambridge, Massachusetts 02142, USA; 4 Department of Urologic Surgery, Vanderbilt University School of Medicine, T-1218 MCN, Nashville, Tennessee 37232-2650, USA; 5 Departments of Biological Sciences and Pathology, Microbiology, and Immunology, Vanderbilt University, T-1218 MCN, Nashville, Tennessee 37232-2650, USA; 6 Department of Cancer Biology, Vanderbilt University School of Medicine, T-1218 MCN, Nashville, Tennessee 37232-2650, USA; 7 Department of Veterans Affairs Medical Center, Nashville, Tennessee 37212-2637, USA

## Abstract

Mechanisms driving persistent airway inflammation in chronic obstructive pulmonary disease (COPD) are incompletely understood. As secretory immunoglobulin A (SIgA) deficiency in small airways has been reported in COPD patients, we hypothesized that immunobarrier dysfunction resulting from reduced SIgA contributes to chronic airway inflammation and disease progression. Here we show that polymeric immunoglobulin receptor-deficient (pIgR^−/−^) mice, which lack SIgA, spontaneously develop COPD-like pathology as they age. Progressive airway wall remodelling and emphysema in pIgR^−/−^ mice are associated with an altered lung microbiome, bacterial invasion of the airway epithelium, NF-κB activation, leukocyte infiltration and increased expression of matrix metalloproteinase-12 and neutrophil elastase. Re-derivation of pIgR^−/−^ mice in germ-free conditions or treatment with the anti-inflammatory phosphodiesterase-4 inhibitor roflumilast prevents COPD-like lung inflammation and remodelling. These findings show that pIgR/SIgA deficiency in the airways leads to persistent activation of innate immune responses to resident lung microbiota, driving progressive small airway remodelling and emphysema.

## Abstract (web summary)

The mechanisms driving lung inflammation and remodelling in chronic obstructive pulmonary disease (COPD) are incompletely understood. Here the authors show that lack of secretory IgA promotes bacterial invasion in small airways, resulting in leukocyte recruitment and a COPD-like phenotype.

## Graphical abstract

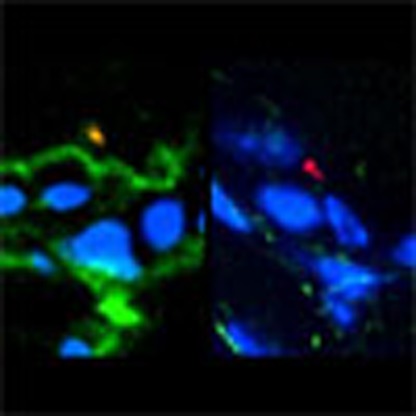

Chronic obstructive pulmonary disease (COPD) is a common smoking-related lung disease defined by fixed obstruction in expiratory airflow and characterized by chronic inflammation, fibrotic remodelling of small airways and emphysematous destruction of lung parenchyma^1^. Fibrotic narrowing of small airways occurs early in the course of COPD and, along with reduced elastic recoil, contributes to airflow obstruction^2^,^3^,^4^. For many years, the predominant hypothesis regarding COPD pathogenesis has been that inhalation of toxic particles and gases, primarily from cigarette smoke (CS), results in oxidant-mediated injury, airway inflammation and disruption of the protease/anti-protease balance favouring lung parenchymal destruction^5^,^6^,^7^. However, this theory does not fully explain the central role of small airways in this disease or continued airway inflammation and disease progression after smoking cessation^8^,^9^.

To protect the lungs from continuous exposure to inhaled irritants, particulates and microorganisms, the airway epithelium forms tight junctions, supports an efficient mucociliary clearance apparatus and maintains a thin airway surface liquid layer that contains a number of components with nonspecific protective activity such as lactoferrin, lysozyme and defensins^10^,^11^,^12^. In addition, epithelial cells support an antigen-specific secretory IgA (SIgA) barrier that covers and protects the airway surface^13^,^14^,^15^. In small airways, polymeric IgA is produced by sub-epithelial plasma cells and transported from the basolateral to apical surface of epithelial cells through binding to the polymeric immunoglobulin receptor (pIgR)^16^,^17^. At the apical surface, pIgR is cleaved to release the secretory component of pIgR joined to polymeric IgA (together forming SIgA) into the airway surface liquid. Through a process known as immune exclusion, SIgA agglutinates airborne antigens and microorganisms, preventing them from activating or injuring airway epithelial cells^14^,^18^,^19^. In patients with COPD, widespread structural abnormalities of the airway epithelium are common and correlate with decreased expression of pIgR and disruption of the SIgA barrier in individual airways^12^,^20^,^21^,^22^,^23^. We have shown that the level of SIgA on the luminal surface of individual small airways correlates inversely with the degree of airway wall remodelling in COPD patients and mean SIgA levels in all small airways across a section of excised lung predicts severity of airflow obstruction^22^. In addition, reduced levels of SIgA are present in bronchoalveolar lavage (BAL) from patients with severe COPD^22^,^24^. To date, however, the contribution of SIgA deficiency to COPD pathogenesis has not been determined. Therefore, we studied mice with genetic deletion of pIgR, which cannot form SIgA on mucosal surfaces. Our studies indicate that pIgR^−/−^ mice develop progressive COPD-like airway and parenchymal remodelling as they age, which results from persistent activation of inflammatory signalling by the lung microbiota, thus pointing to a causative role for SIgA deficiency in persistent inflammation and disease progression in COPD.

## Results

### Lung inflammation and remodelling in pIgR^−/−^ mice

**Figure 1:**
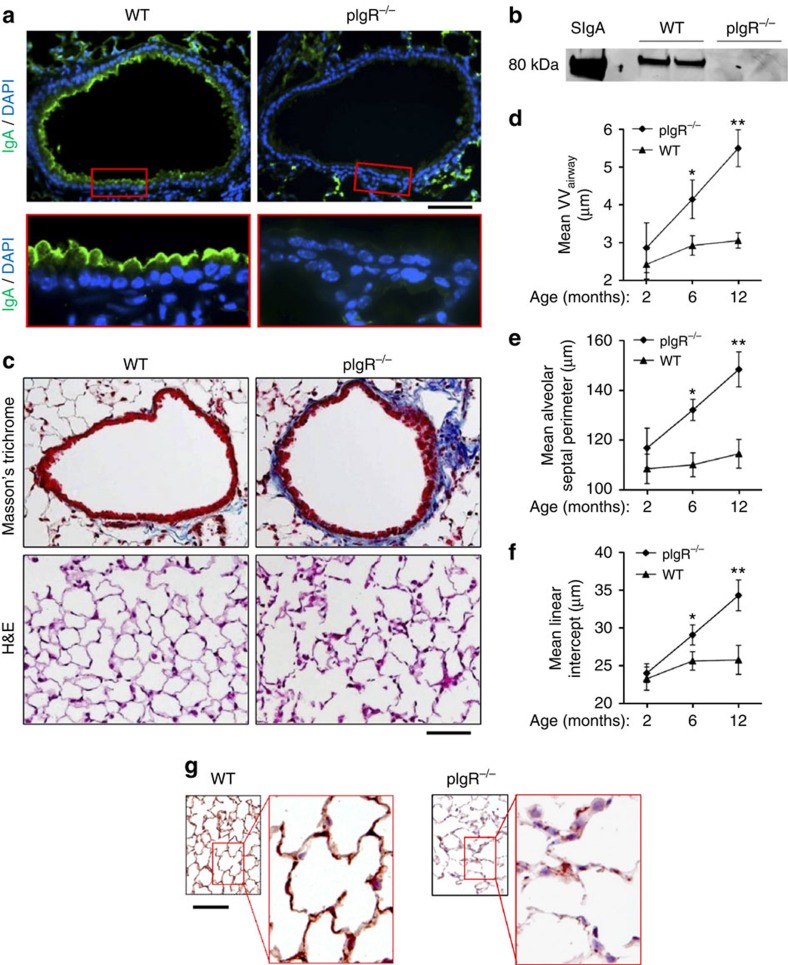
pIgR^−/−^ mice develop progressive COPD-like small airway and parenchymal remodelling. (**a**) Immunofluorescence staining for IgA (green) showing SIgA on the epithelial surface of a small airway from a WT mouse and no detectable SIgA on the airway surface of a pIgR^−/−^ mouse (original magnification, × 200 and × 1,000 (insets)). Scale bar, 50 μm. (**b**) Western blotting for secretory component in BAL fluid from WT and pIgR^−/−^ mice. SIgA from human colostrum was used as a positive control. (**c**) Representative images of small airway remodelling (Masson's trichrome, original magnification, × 200) and emphysema (haematoxylin and eosin (H&E), original magnification, × 200) in a 12-month-old pIgR^−/−^ mouse compared with a WT control. Scale bar, 50 μm. (**d**–**f**) Morphometric analysis showing increased wall thickness (VV_airway_), mean alveolar septal perimeter length and mean linear intercept in pIgR^−/−^ and age-matched WT littermate controls at the indicated ages. Five to ten mice per group; *P<0.01 compared with 2-month-old pIgR^−/−^ mice and age-matched WT controls; **P<0.001 compared with all other groups (two-way analysis of variance (ANOVA)). (**g**) Immunostaining for elastin in 12-month-old WT and pIgR^−/−^ mice shows reduction and fragmentation of elastin in inter-alveolar septa in a pIgR^−/−^ mouse compared with the intact elastin network in a WT mouse (original magnification, × 100 and × 1,000 (insets)).

We obtained pIgR^−/−^ mice (C57BL/6 background)^25^,^26^ and performed immunofluorescence microscopy to show that SIgA was not detectable on the airway surface (Fig. 1a). In addition, western blottings for secretory component from BAL fluid confirmed a lack of SIgA in the airways of pIgR^−/−^ mice (Fig. 1b). Although these mice appeared healthy at birth and demonstrated no histopathologic changes in the lungs compared with wild-type (WT) littermate controls at 2 months of age, pIgR^−/−^ mice developed COPD-like changes with fibrotic small airway remodelling and emphysematous destruction of the lung parenchyma by 6 months of age, which continued to worsen in 12-month-old mice (Fig. 1c–f). Despite the presence of airway wall remodelling in pIgR^−/−^ mice, airway epithelial structure appeared intact without evidence of goblet cell hyperplasia or stratification. Similar to COPD patients^27^,^28^, ageing pIgR^−/−^ mice displayed fragmentation and degradation of the elastin network in alveolar walls and around small airways (Fig. 1g). Importantly, unlike other genetic models of COPD^29^, the lack of COPD-like changes in 2-month-old (young adult) pIgR^−/−^ mice indicates that this phenotype is not related to developmental defects resulting from *in utero* pIgR deficiency.

**Figure 2:**
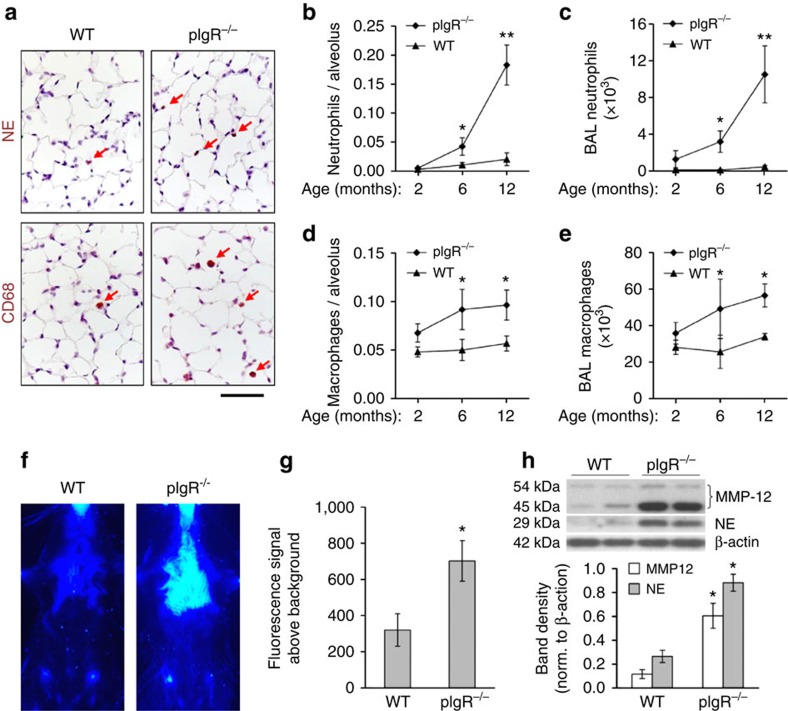
Lung inflammation progresses with age in pIgR^−/−^ mice. (**a**) Representative immunostains for neutrophils using antibodies to neutrophil elastase (NE) or macrophages using antibodies to CD68 in 12-month-old WT and pIgR^−/−^ mice. Positive cells are stained brown (indicated by red arrows) (original magnification, × 200). Scale bar, 50 μm. (**b**–**e**) Neutrophil (NE^+^) and macrophage (CD68^+^) counts in lungs of pIgR^−/−^ and age-matched WT littermate controls at the indicated ages, and neutrophil and macrophage counts in BAL fluid. Five to seven mice per group; *P<0.05 compared with 2-month-old pIgR^−/−^ mice and age-matched WT mice; **P<0.01 compared with all other groups (two-way analysis of variance (ANOVA)). (**f**) Representative image of folate-PEG-Cy5-derived chest fluorescence 4 h after intravenous probe injection in 12-month-old WT and pIgR^−/−^ mice. (**g**) Photon emission from the chest normalized to background before injection of probe. Three to four mice per group; *P<0.05 (Student's *t*-test). (**h**) Western blotting and densitometry for MMP-12 (two bands at 45 and 54 kDa) and NE (29 kDa) in lung tissue from 12-month-old WT and pIgR^−/−^ mice. Band densities of MMP-12 and NE were normalized to β-actin. Six mice per group; *P<0.01 compared with WT mice (Student's *t*-test).

After identifying COPD-like changes in the lungs of pIgR^−/−^ mice, we quantified inflammatory cells in the lungs. At 2 months of age, WT and pIgR^−/−^ mice showed similar numbers of neutrophils and macrophages in lung parenchyma and in BAL; however, by 6 and 12 months of age, pIgR^−/−^ mice had a remarkable increase in inflammatory cells compared with 2-month-old mice and with age-matched WT controls (Fig. 2a–e). Macrophage accumulation in the lungs of pIgR^−/−^ mice was similar at 6 and 12 months of age, but the neutrophil influx continued to increase between 6 and 12 months of age. In addition, lymphocytes were found to be increased in lungs of pIgR^−/−^ mice compared with WT controls. Total lymphocyte counts in BAL were 789±135 in 12-month-old WT mice compared with 3027±287 in 12-month-old pIgR^−/−^ mice (*P*<0.001).

We recently developed a non-invasive *in vivo* molecular imaging technique that uses a fluorescent probe (folate-PEG-Cy5) to identify activated macrophages based on the expression of folate receptor-β^30^. As shown in Fig. 2f,g, increased fluorescent signal was detected over the lungs of 12-month-old pIgR^−/−^ mice compared with age-matched WT controls, indicating an increase in activated macrophages in the lungs of these mice. Interestingly, we observed no difference in the fluorescent signal over the abdomen of pIgR^−/−^ mice, suggesting that increased macrophage activation was limited to lungs.

Activated macrophages and neutrophils can produce matrix metalloproteinase (MMP)-12 and neutrophil-derived elastase (NE), respectively, which have been linked to emphysematous remodelling^31^,^32^,^33^,^34^. Therefore, we measured MMP-12 and NE in lung homogenates and found significantly increased levels of both these enzymes in 12-month-old pIgR^−/−^ mice compared with age-matched WT mice (Fig. 2h and Supplementary Fig. 1). Together, these data indicate that pIgR^−/−^ mice develop a persistent inflammatory and destructive environment in the lungs as they age, probably contributing to the COPD phenotype observed in these mice.

### Bacterial invasion and NF-κB activation in pIgR^−/−^ mice

**Figure 3:**
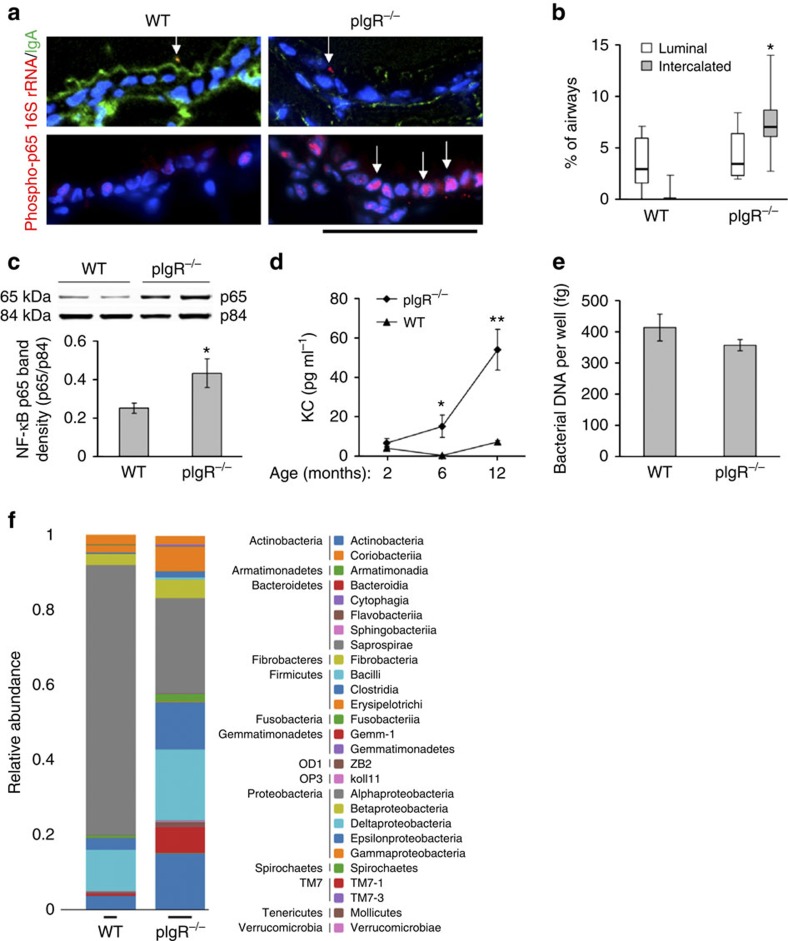
Bacterial invasion and NF-κB activation in airways of pIgR^−/−^ mice. (**a**) Immunofluorescent detection of bacteria (fluorescent *in situ* hybridization (FISH) probe for 16S rRNA, red, top panels) or NF-κB (phospho-p65 (Ser276, red, bottom panels), IgA (green, top panels) and DAPI (blue) from 12-month-old WT and pIgR^−/−^ mice (original magnification, × 1,000). In WT mice, bacteria with bound SIgA were identified within the airway lumen (yellow on merged image, identified by arrow), whereas bacteria intercalated within the epithelium were identified in pIgR^−/−^ mice (red, identified by arrow). Bottom panels show phopsho-p65 localized to nuclei (arrows). Scale bar, 50 μm. (**b**) Box and whisker plot (showing median, 25th–75th percentile and range) for intraepithelial (intercalated) and luminal bacteria in airways of 12-month-old WT and pIgR^−/−^ mice as identified by FISH for bacterial DNA. Seven mice per group; *P<0.001. (**c**) Western blotting and densitometry for p65 component of NF-κB (normalized to p84) in nuclear protein extracts from lungs of 12-month-old WT and pIgR^−/−^ mice. Six mice per group; *P<0.05 (Student's *t*-test). (**d**) KC protein levels in BAL fluid. Six mice per group; *P<0.05 compared with 2-month-old pIgR^−/−^ mice and age-matched WT controls; **P<0.05 compared with all other groups (two-way analysis of variance (ANOVA)). (**e**) Total bacterial DNA was quantified from lung tissue in WT and pIgR^−/−^ mice using quantitative PCR (qPCR) and primers specific for V1 region of prokaryotic 16S rRNA. Ten mice per group. (**f**) Distribution of bacterial phyla and classes in lung tissue as determined by 16S sequencing from lungs of 12-month-old WT and pIgR^−/−^ mice (three mice studied per group but only two WT mice had sufficient bacterial DNA amplification for detailed analysis).

As loss of mucosal immunity in pIgR-deficient mice results in chronic inflammation in the lungs, we wondered whether lack of SIgA could render small airways more susceptible to invasion by airway bacteria with subsequent activation of inflammatory signalling in epithelial cells. Therefore, we performed fluorescent *in situ* hybridization on lung sections from WT and pIgR^−/−^ mice using probes specific for the conserved portion of the bacterial gene encoding 16S ribosomal RNA (Fig. 3a top panels). Although a significant proportion of airways in pIgR^−/−^ mice showed bacteria localized within the airway epithelium (between the apical epithelial border and basement membrane), this was almost never observed in WT airways. In contrast, the percentage of airways with bacteria present in the airway lumen did not differ between WT and pIgR^−/−^ mice (Fig. 3b). These findings suggest that an impaired mucosal immune barrier in pIgR-deficient mice allows migration of colonizing airway bacteria across the apical epithelial border.

Bacteria can initiate innate immune signalling in the epithelium through activation of Toll-like receptors, leading to activation of the nuclear factor-κB (NF-κB) pathway. To investigate whether this pathway was activated in pIgR^−/−^ mice, we performed fluorescent immunostaining for an activated form of the p65(RelA) component of NF-κB (phosphoserine 276) (refs 35, 36, 37) (Fig. 3a bottom panels). Although detection of phospho-p65 (Ser276) staining in nuclei of airway epithelial cells was rare in WT mice, a remarkable upregulation of phospho-p65 was detected in lungs of 6- and 12-month-old pIgR^−/−^ mice. We also evaluated NF-κB activation in lung tissue by western blotting and found increased p65 in nuclear protein extracts from pIgR^−/−^ mice compared with age-matched WT controls, further supporting increased NF-κB activation in these mice (Fig. 3c and Supplementary Fig. 2). In addition, we identified a significant increase in the concentration of the NF-κB-dependent chemokine keratinocyte chemoattractant (KC) in BAL fluid from pIgR^−/−^ mice compared with age-matched WT mice (Fig. 3d). The combination of bacterial localization within the airway epithelium and increased epithelial NF-κB activation in pIgR^−/−^ mice supports the conclusion that loss of surface SIgA allows colonizing bacteria to penetrate the epithelial barrier and activate inflammatory signalling.

To determine whether pIgR deficiency alters the density or composition of the lung microbiome, we analysed bacterial abundance and taxonomy in the lungs of age-matched WT and pIgR^−/−^ mice. First, we measured total bacterial DNA by quantitative PCR targeting the V1/V2 portion of bacterial 16S rRNA in 9-month-old mice and found no difference in total bacterial burden in the lungs of WT and pIgR^−/−^ mice (Fig. 3e). Subsequently, we used high-throughput amplicon sequencing to evaluate microbial communities in whole lung tissue from 6-month-old WT and pIgR^−/−^ mice. As has been reported previously^38^, Proteobacteria and Firmicutes were the most common bacterial phyla present in the lungs of both groups of mice (Fig. 3f). Compared with WT mice, pIgR^−/−^ mice had a twofold increase (400 versus 194) in the number of detected operational taxonomic units (OTUs) (Fig. 3f and Supplementary Fig. 3). Analysis by Random Forests, a supervised machine learning technique, was used to classify microbial taxa that discriminate the mouse genotypes^39^. Ten OTUs that best discriminate the genotypes are shown in Supplementary Fig. 4.

### SIgA modulates the acute inflammatory response to NTHi

**Figure 4:**
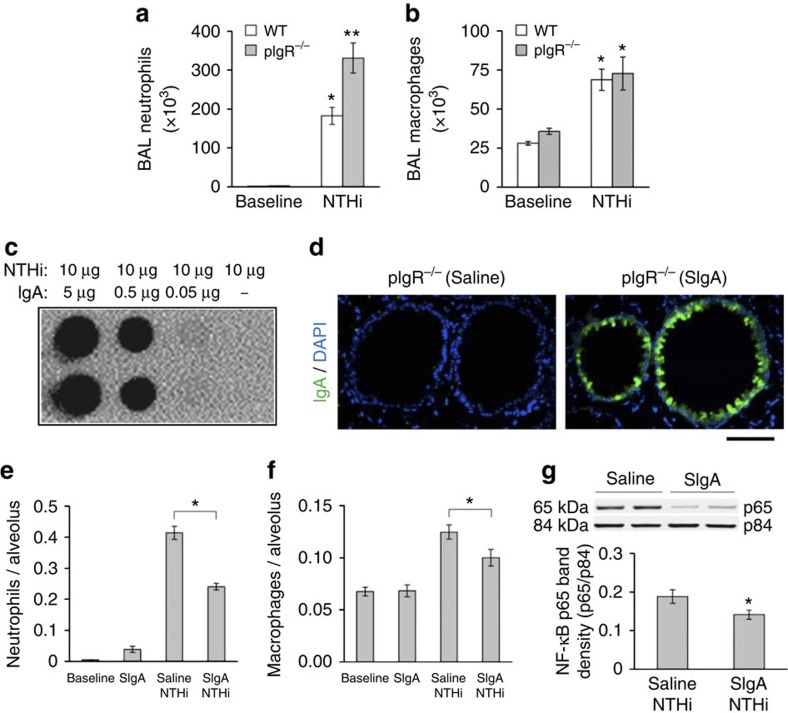
SIgA modulates the acute inflammatory response to NTHi *in vivo*. (**a**,**b**) Neutrophil and macrophage counts in BAL fluid from 2-month-old WT and pIgR^−/−^ mice 24 h after aerosolization of NTHi lysate (10 mg). Six to eight mice per group; *P<0.01 compared with untreated mice (baseline), **P<0.01 compared with WT mice treated with NTHi (Student's *t*-test). (**c**) Dot-blot assay demonstrating protein binding between NTHi lysates and human SIgA from colostrum. (**d**) Immunofluorescent detection of human SIgA (green) in the lungs of pIgR^−/−^ mouse 1 h after i.t. delivery of SIgA or vehicle (normal saline) (original magnification, × 200). Scale bar, 50 μm. (**e**,**f**) Parenchymal neutrophil and macrophage counts 24 h after aerosol delivery of NTHi lysate to 2-month-old pIgR^−/−^ mice pretreated with i.t. SIgA (50 μl of 0.34 mg ml^−1^ solution) or vehicle (normal saline). Macrophage and neutrophil numbers were quantified by immunostaining for CD68 or NE, respectively. Five to six mice per group; *P<0.05 compared with mice pretreated with saline followed by NTHi (Student's *t*-test). (**g**) Western blotting and densitometry for p65 component of NF-κB (normalized to p84) in lung nuclear protein extracts from 2-month-old pIgR^−/−^ mice pretreated with i.t. SIgA or normal saline 1 h before NTHi nebulization and harvested 24 h later. Six mice per group; *P<0.05 (Student's *t*-test).

To investigate whether pIgR deficiency directly alters the inflammatory response to bacterial products, we treated 2-month-old WT or pIgR^−/−^ mice with lysates prepared from non-typeable *Haemophilus influenzae* (NTHi). Although NTHi is not a mouse pathogen it was selected for study, because it is the most common bacterium identified in the respiratory tract of patients with COPD^40^,^41^. Compared with WT mice, pIgR^−/−^ mice treated with aerosolized NTHi lysate had increased inflammation as determined by neutrophil influx after 24 h (Fig. 4a,b). To determine whether exogenous SIgA could mitigate inflammation induced by NTHi lysates, we obtained SIgA from pooled human colostrum, showed that this SIgA binds to proteins in NTHi lysates and delivered SIgA into the lungs of pIgR^−/−^ mice by intratracheal (i.t.) injection (Fig. 4c,d). At 24 h after exposure to aerosolized NTHi lysates, pIgR^−/−^ mice treated with i.t. SIgA showed a remarkable reduction in NTHi-induced lung inflammation and NF-κB activation compared with pIgR^−/−^ mice treated with vehicle (Fig. 4e–g and Supplementary Fig. 5), indicating that the presence of SIgA limits the inflammatory response to bacterial antigens in the lungs.

### Bacteria drive the COPD-like phenotype in pIgR^−/−^ mice

**Figure 5:**
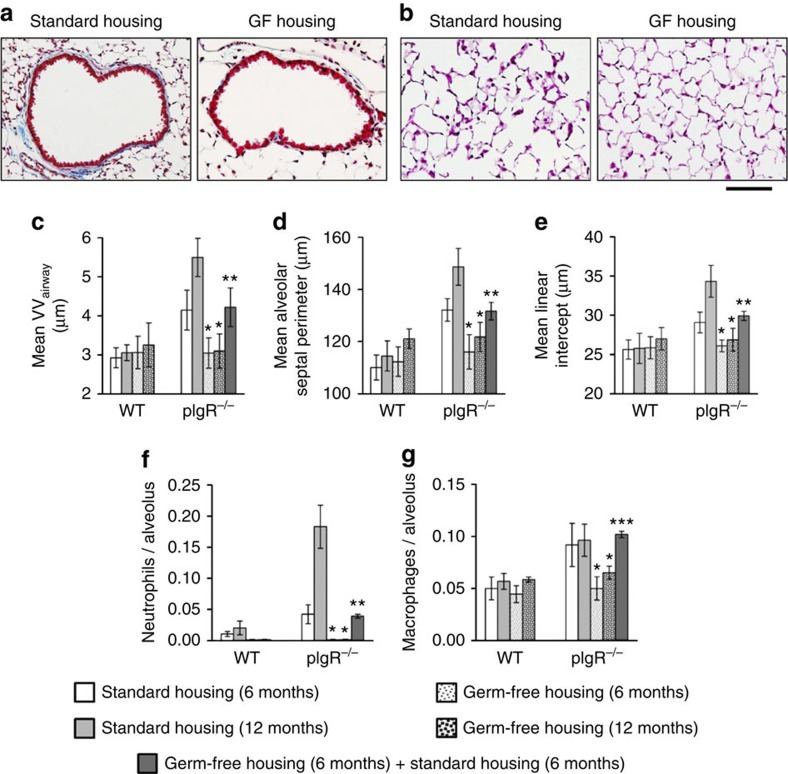
Germ-free pIgR^−/−^ mice are protected from COPD-like lung remodelling. (**a**) Representative images of small airway remodelling (Masson's trichrome, original magnification, × 200) and emphysema (**b**) (haematoxylin and eosin (H&E), original magnification, × 200) in a 6-month-old pIgR^−/−^ mouse in standard housing compared with a 6-month-old pIgR^−/−^ mouse housed in germ-free conditions. Scale bar, 50 μm. (**c**–**e**) Morphometric analysis of airway wall thickness (VV_airway_), alveolar septal perimeter length and mean linear intercept in 6- and 12-month-old WT and pIgR^−/−^ mice maintained in standard housing, germ-free housing or 6 months of germ-free housing followed by 6 months of standard housing as indicated. (**f**,**g**) Parenchymal neutrophil (NE+) and macrophage (CD68+) counts in lungs of 6- and 12-month-old WT and pIgR^−/−^ mice maintained in standard housing, germ-free housing or a combination of both as indicated. Six to seven mice per group; *P<0.001 compared with all other groups, **P<0.001 compared with age-matched WT controls housed in standard conditions (two-way analysis of variance (ANOVA)).

Given our observation that pIgR-deficient mice have increased bacterial invasion across the mucosal surface of the airways, changes in microbial composition and a heightened inflammatory response, we postulated that endogenous bacterial flora in pIgR^−/−^ mice could be responsible for driving persistent inflammation and COPD-like remodelling in the lungs of these mice. To test this concept, germ-free pIgR^−/−^ and WT mice (C57BL/6 background) were generated at the National Gnotobiotic Rodent Resource Center and maintained in sterile conditions. In contrast to pIgR^−/−^ mice maintained in standard housing, 6-month-old germ-free pIgR^−/−^ mice were completely protected from small airway remodelling and emphysema (Fig. 5a–e). Although the COPD-like remodelling progressed from 6 to 12 months of age in pIgR^−/−^ mice housed in standard conditions, no evidence of small airway remodelling or emphysema was observed in germ-free pIgR^−/−^ mice, even at 12 months of age. Neutrophils were essentially undetectable in the alveolar parenchyma from germ-free pIgR^−/−^ and WT mice, and macrophage counts in germ-free pIgR^−/−^ mice were reduced to levels similar to that of WT mice (with standard or sterile housing) (Fig. 5f,g). To investigate the impact of reconstituting the microbiome in adult pIgR-deficient mice, we removed a cohort of mice from germ-free conditions at 6 months of age and housed them in standard conditions for 6 months. As shown in Fig. 5c–g, 12-month-old pIgR-deficient mice (which were maintained in standard housing for 6 months) demonstrated similar levels of airway wall remodelling, emphysema and inflammation to 6-month-old pIgR^−/−^ mice raised in standard housing. Cumulatively, these results implicate airway bacteria as the primary driver of inflammation and COPD-like histopathologic changes in pIgR^−/−^ mice.

### Roflumilast blocks COPD progression in pIgR^−/−^ mice

**Figure 6:**
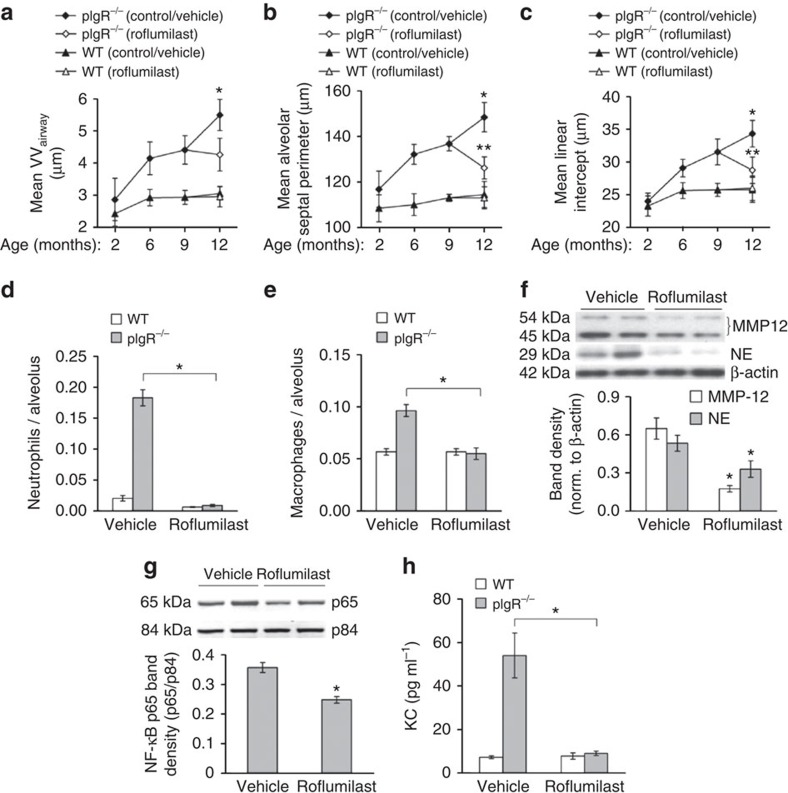
Roflumilast blocks inflammation and COPD-like lung remodelling in pIgR^−/−^ mice. (**a**–**c**) Morphometric analysis showing small airway wall thickness (VV_airway_), mean alveolar septal perimeter length and mean linear intercept at the indicated ages in pIgR^−/−^ and WT mice treated with roflumilast or vehicle from 9 to 12 months of age. Five to ten mice per group; *P<0.01 compared with 12-month-old pIgR^−/−^ mice treated with roflumilast, **P<0.05 compared with 6- and 9-month-old pIgR^−/−^ mice (two-way analysis of variance (ANOVA)). (**d**,**e**) Parenchymal neutrophil and macrophage counts in 12-month-old WT and pIgR^−/−^ mice treated for 3 months with roflumilast or vehicle. Six to seven mice per group; *P<0.01 (macrophages) or *P*<0.001 (neutrophils) compared with pIgR^−/−^ mice treated with vehicle (Student's *t*-test). (**f**) Western blotting and densitometry for MMP-12 and NE in lung tissue from 12-month-old pIgR^−/−^ mice treated with roflumilast or vehicle. Band densities of MMP-12 and NE were normalized to β-actin. Six mice per group; *P<0.05 (Student's *t*-test). (**g**) Western blotting and densitometry for p65 component of NF-κB (normalized to p84) in lung nuclear protein extracts from 12-month-old pIgR^−/−^ mice treated with roflumilast or vehicle. Six mice per group; *P<0.01 (Student's *t*-test). (**h**) KC protein levels in BAL fluid from 12-month-old WT or pIgR^−/−^ mice treated with roflumilast or vehicle. Six mice per group; *P<0.05 compared with pIgR^−/−^ mice treated with vehicle (Student's *t*-test).

Next, to investigate whether progressive small airway remodelling and emphysema in pIgR^−/−^ mice occur in response to bacteria-induced inflammation, we used the anti-inflammatory drug roflumilast, which inhibits phosphodiesterase-4. Roflumilast is FDA approved for use in COPD patients and has been shown to reduce inflammation in murine models of COPD^42^,^43^,^44^,^45^. For these studies, 9-month-old WT or pIgR^−/−^ mice were treated daily by oral gavage with 100 μg of roflumilast (5 μg g^−1^) or vehicle (4% methylcellulose, 1.3% PEG400) for 3 months and lungs were harvested at 12 months of age. Unlike pIgR^−/−^ mice treated with vehicle, mice treated with roflumilast had no progression of small airway wall remodelling after starting treatment (Fig. 6a). Strikingly, 12-month-old pIgR^−/−^ mice treated with roflumilast had reduced indices of emphysema compared with 9-month-old pIgR^−/−^ mice, indicating that roflumilast not only blocks progression of emphysema in this model but apparently facilitates some resolution of the emphysematous destruction of lung parenchyma (Fig. 6b,c). Similar to mice housed in germ-free conditions, WT and pIgR^−/−^ mice treated with roflumilast had very few neutrophils in the lung parenchyma (Fig. 6d) and macrophage numbers were equivalent to vehicle-treated WT mice (Fig. 6e). Consistent with decreased inflammation, roflumilast treatment resulted in reduced MMP-12 and NE in lungs of pIgR^−/−^ mice (Fig. 6f and Supplementary Fig. 6). In addition, NF-κB activation and KC expression were reduced in lungs of roflumilast-treated pIgR^−/−^ mice compared with vehicle-treated pIgR^−/−^ mice (Fig. 6g,h and Supplementary Fig. 7). Together, these data indicate that persistent bacterial-derived inflammation propels COPD-like remodelling in pIgR^−/−^ mice.

### Effects of CS in pIgR^−/−^ and WT mice

**Figure 7:**
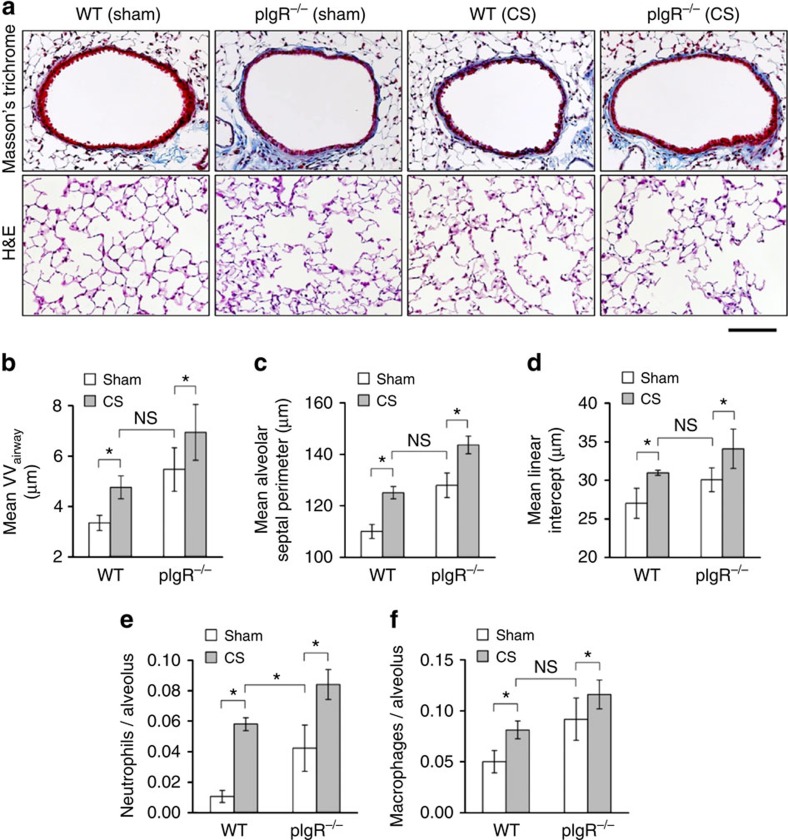
CS treatment increases airway remodelling and emphysema in pIgR^−/−^ mice. (**a**) Representative images of small airway remodelling (Masson's trichrome, original magnification, × 200X and emphysema (haematoxylin and eosin (H&E), original magnification, × 200) in WT and pIgR^−/−^ mice treated twice daily with mainstream CS or sham control (filtered air) for 6 months (between 2 and 8 months of age). Scale bar, 50 μm. (**b**–**d**) Morphometric analysis of small airway wall thickness (VV_airway_), mean alveolar septal perimeter length and mean linear intercept length. Fve mice per group; *P<0.01 compared with WT mice treated with sham; **P<0.01 compared with all other groups (two-way analysis of variance (ANOVA)). (**e**,**f**) Parenchymal neutrophil and macrophage counts in lung tissue from WT or pIgR^−/−^ mice. Five mice per group; *P<0.01 compared with WT mice treated with sham; **P<0.01 compared with all other groups (two-way ANOVA).

To determine how spontaneous COPD-like remodelling in pIgR^−/−^ mice compares with long-term CS exposure, we treated 2-month-old WT and pIgR^−/−^ mice with mainstream CS twice daily for 6 months, according to a protocol previously shown to induce emphysema^46^. WT mice treated with CS developed a similar degree of small airway wall remodelling and emphysema compared with sham-treated pIgR^−/−^ mice; however, CS exposure worsened COPD-like remodelling in pIgR^−/−^ mice (Fig. 7a–d). No evidence of structural airway epithelial changes, including goblet cell hyperplasia or stratification, or development of lymphoid aggregates or tertiary lymphoid follicles was present in any group of mice (with or without CS treatment). Compared with age-matched, sham-treated WT controls, increased inflammatory cells (neutrophils and macrophages) were observed in CS-treated WT mice and pIgR^−/−^ mice with or without CS treatment (Fig. 7e,f). The only observed difference between CS-treated WT mice and sham-treated pIgR^−/−^ mice was a mild increase in neutrophil influx in the CS-treated WT group. In this study, the highest degree of remodelling and inflammation was present in CS-treated pIgR^−/−^ mice, indicating an additive effect between pIgR^−/−^ deficiency and CS exposure in this model.

## Discussion

This work elucidates an important role for the SIgA immune system in maintaining homeostasis in the lungs by showing that disruption of this first line of mucosal host defense leads to persistent activation of innate immunity, which is normally reserved as a second line of host defense. Chronic innate immune activation, in turn, drives tissue injury and progressive lung remodelling. Mice with a defective SIgA immune system in the lungs due to pIgR deficiency develop a pattern of small airway and parenchymal remodelling that recapitulates pathological changes seen in human COPD. This phenotype worsens with ageing, indicating that injury and remodelling due to pIgR/SIgA deficiency are additive and progressive. pIgR^−/−^ mice develop increased bacterial invasion into small airway walls, resulting in epithelial cell NF-κB activation, leukocyte recruitment and upregulation of MMP-12 and NE expression. Exogenous SIgA replacement reduces lung inflammation in response to bacterial lysates, thus showing a direct anti-inflammatory effect of SIgA in the lungs. Chronic lung inflammation in pIgR^−/−^ mice is abrogated by germ-free housing, implicating bacterial invasion into SIgA-deficient airways as the central driver of inflammation in pIgR^−/−^ mice. Long-term treatment with the anti-inflammatory drug roflumilast blocks progressive small airway wall remodelling and partially reverses emphysematous changes in SIgA-deficient mice with established disease, showing that inflammatory cells and mediators are responsible for COPD-like remodelling. In addition, we found that long-term CS treatment of WT mice resulted in a COPD-like phenotype that was similar in magnitude to the spontaneous phenotype in age-matched pIgR^−/−^ mice. Together with prior publications showing widespread airway surface SIgA deficiency in COPD patients^20^,^22^, our data support the concept that reduced pIgR expression and acquired SIgA deficiency in the airways of humans contributes to chronic inflammation and disease progression in COPD.

It has long been appreciated that remodelling of small resistance airways is an important determinant of airflow obstruction^2^,^3^. Our data help to explain the central role of small airways in COPD. We propose that leukocytes recruited to small airways with acquired SIgA deficiency produce proteases that damage the airway walls, resulting in fibrotic remodelling and, ultimately, airflow obstruction. In addition, products of activated leukocytes including MMP-12 and NE can cause destruction of elastin fibres and other components of the interalveolar septum adjacent to these small airways, leading to centrilobular emphysema. Although reduced pIgR expression and SIgA levels in COPD patient airways correlates with abnormal epithelial differentiation^22^, the mechanism responsible for reduced pIgR expression and acquired SIgA deficiency in COPD is uncertain and is an important area for further study. Once individual small airways develop pIgR/SIgA deficiency, our data suggest that inflammation may become self-perpetuating, potentially explaining the persistence of airway inflammation in COPD patients after smoking cessation^8^,^9^.

Our studies indicate that the lung microbiota drives chronic lung inflammation and remodelling in the setting of defective mucosal immunity. Although pIgR^−/−^ mice did not show evidence for widespread bacterial overgrowth in the lungs, these mice have increased bacterial invasion across the apical epithelial surface of small airways. In initial studies, we noted an expansion in Alphaproteobacteria and higher community diversity in pIgR^−/−^ mice. Our analysis suggests that a number of OTUs may discriminate between WT and pIgR^−/−^ mice; however, expansion of these findings using a much larger cohort will be required for more definitive conclusions. These data do not exclude the possibility that systemic effects of an altered gut microbiome in pIgR^−/−^ mice could also contribute to the development of lung remodelling in pIgR-deficient mice. Future studies should determine the composition of the murine lung and gut microbiome before and after the development of lung remodelling in WT and pIgR^−/−^ mice, and investigate whether the microbiome of pIgR^−/−^ mice is intrinsically more inflammatory than that of WT mice.

In addition to bacteria, viruses and environmental antigens could also have an impact on chronic inflammation and COPD-like remodelling in the setting or mucosal immune deficiency. In intestinal epithelial cells, pIgR is upregulated by double-stranded RNA via TLR3, consistent with a role in protection against viruses^47^. We have previously shown that SIgA-deficient airways have increased expression of cytomegalovirus late antigen and Epstein-barr virus (EBV) latent membrane protein compared with SIgA-replete airways from the same individual^22^. Although colonizing bacteria appear to be the most important driver of COPD-like lung remodelling in pIgR^−/−^ mice housed in the protected environment of our animal care facility, environmental antigens and viruses could also be important drivers of inflammation and progressive disease in COPD patients with acquired SIgA deficiency in small airways.

We found that roflumilast blocks inflammatory cell recruitment and prevents small airway wall remodelling and emphysema that develop in response to pIgR/SIgA deficiency. Roflumilast reduces inflammation in the lungs of COPD patients and is FDA approved for use in patients with severe COPD^48^, where it has been shown to reduce disease exacerbations. Our studies, however, suggest that roflumilast could have a disease-modifying effect in COPD and may be beneficial in patients with less advanced disease.

In our models, pIgR deficiency and long-term CS exposure showed a similar degree of inflammation and COPD-like remodelling in the lungs; however, the combination of pIgR deficiency and cigarette smoking appeared to have an additive effect on small airway wall remodelling and emphysema. This latter finding suggests that the effects of CS and pIgR deficiency are independent in this model. We postulate that the lack of interaction between CS and pIgR/SIgA deficiency may be explained by a lack of structural remodelling of the airway epithelium (for example, goblet cell hyperplasia or stratification) in our CS model, which may be necessary for repressing pIgR expression. Ganesan *et al.*^49^ showed that the combination of chronic CS exposure and bacterial challenge can induce goblet cell hyperplasia, as well as tertiary lymphoid follicles, in mice. Studies using combined stimuli that cause structural remodelling of airway epithelium in mice may be necessary to study the effects of acquired pIgR/SIgA deficiency in mouse models.

An implication of our findings is that patients with genetic IgA deficiency, the most common immunodeficiency in humans^50^, might be at increased risk for the development of COPD. To our knowledge, no large epidemiologic studies have evaluated whether IgA deficiency is associated with an increased risk for COPD. However, patients with genetic IgA deficiency have normal or even increased levels of IgM^51^,^52^, which may compensate for lack of SIgA in small airways. In contrast, reduced pIgR expression, which is present in airways of COPD patients^22^, limits transport of both dimeric IgA and IgM to the airway surface. Nonetheless, future studies to investigate the incidence and progression of obstructive lung disease in IgA-deficient individuals could be informative.

In summary, our studies demonstrate that surface SIgA deficiency in small airways of pIgR^−/−^ mice leads to persistent activation of innate immune responses to resident lung microbiota and generation of a phenotype that resembles important aspects of human COPD. Our findings highlight a critical role for pIgR/SIgA in maintenance of the immune-barrier function of the airway epithelium and could explain several key aspects of COPD pathogenesis, including the central role of small airways and persistent airway inflammation even after smoking cessation. Therapeutic strategies that restore normal immune barrier function to the small airways or deplete the airways of bacteria may be of therapeutic benefit to patients with COPD.

## Methods

### Animal model

pIgR^−/−^ mice, backcrossed onto a C57Bl/6 background for a minimum of eight generations^25^,^26^, were obtained from the Mutant Mouse Resource Research Center at the University of Missouri. WT and pIgR^−/−^ mice were housed in standard microisolator cages in a centralized animal care facility and provided food and water *ad libitum*. Germ-free mice pIgR^−/−^ were surgically derived by sterile embryo transfer and maintained in sterile flexible film Trexler isolators at the National Gnotobiotic Rodent Resource Center (University of North Carolina School of Medicine, Chapel Hill, NC). Within Trexler isolators, mice were housed in standard microisolator cages with sterilized bedding and provided with sterilized rodent chow and water *ad libitum*. Sterility was documented on a monthly basis by faecal Gram stain, aerobic and anaerobic cultures, and PCR for 16S rRNA of the faeces and bedding. For selected mice, sterility of faeces was also documented by Gram stain and cultures at the time of necropsy. For all experiments, male and female mice were killed from age 2–12 months and compared with age-matched WT mice as indicated. For the microbiome and germ-free experiments, WT and germ-free mice were housed together. For all other experiments, WT and pIgR^−/−^ were housed separately. All procedures involving mice were approved by the Institutional Care and Use Committee of Vanderbilt University.

### *In vivo* treatments

WT or pIgR^−/−^ mice were exposed to mainstream CS from two 3R4F cigarettes once daily for 2 weeks, followed by four 3R4F cigarettes twice daily until killing at 8 months. Cigarettes were smoked sequentially, one 5-s puff per minute for a total of seven puffs per cigarette, using a nose-only exposure system (*inExpose*, SCIREQ, Montreal, CA). Control animals were housed in identical nose-only cages but were exposed to filtered air only. For NTHi nebulization studies, mice were placed in a whole-body nebulization chamber (*inExpose*, SCIREQ) and exposed to 10 mg of aerosolized NTHi lysate delivered by a 5 l min^−1^ pump. Control animals were treated with aerosolized PBS. For the SIgA pretreatment experiment, 1 h before nebulization, 50 μl of a 0.34 mg ml^−1^ solution of IgA from pooled human colostrum (Sigma-Aldrich, St Louis, MO) or 50 μl sterile PBS was administered i.t. after intubation of isofluorane-anaesthetized mice.

### Roflumilast administration

For studies using roflumilast, 200 μl of 0.5 mg ml^−1^ suspension of roflumilast or vehicle (4% methylcellulose, 1.3% PEG400 and ∼5 μg drug per mg animal weight) was administered by oral gavage once daily, 5 days a week for the duration of treatment. The roflumilast suspension was freshly prepared each week and stored at 4 °C.

### Preparation of NTHi lysates

NTHi strain 1479 (a gift from Dr Brahm Segal, University of Buffalo) was grown overnight on chocolate agar plates (Hardy Diagnostics, Santa Maria, CA) and then used to inoculate 20 ml of brain–heart infusion media (Sigma-Aldrich) containing 10 μg ml^−1^ NAD and 10 μg ml^−1^ Hemin (both from Sigma-Aldrich). The culture was incubated for 4 h at 36 °C with constant shaking and then used to inoculate an additional 200 ml of liquid media. After another 4 h of growth, bacteria were pelleted, boiled for 1 h, sonicated twice for 1 min each and filtered through a 0.22-μM polyethylsulfone filter (EMD Millipore, Darmstadt, Germany) using 1 ml of added PBS for each pellet generated from 50 ml of liquid culture. Protein concentration was adjusted to 1.5 mg ml^−1^ in PBS using Bradford assay (Pierce, Rockford, IL).

### Lung harvest technique and BAL

After perfusion with normal saline, the left lung was isolated, removed and flash-frozen in liquid nitrogen, whereas the right lung was inflated using a 25-cm pressure column containing 10% neutral buffered formalin. The frozen left lungs were stored at −80 °C and used for protein analyses. The right lungs were fixed overnight in 10% formalin and then embedded in paraffin for histological analyses. BAL was performed using two 500-μl aliquots of sterile PBS. Fluid was combined and centrifuged at 400 *g* for 10 min, to separate cells from the supernatant. Supernatant was stored at −80 °C and then used for cytokine and chemokine measurements. Separate animals were used for histological analyses and BAL.

### Histology and immunohistochemistry

Five-micrometre serial sections were cut from each tissue specimen and haematoxylin and eosin, and Masson trichrome staining was performed. Additional serial sections were used for immunostaining with rabbit polyclonal anti-IgA (PAB9360, Abnova, Taipei City, Taiwan; 1:100) to detect SIgA on airway epithelial surface, rabbit polyclonal anti-neutrophil elastase (ab68672, Abcam; 1:200) to detect neutrophils, rabbit polyclonal anti-CD68 (ab125512, Abcam; 1:200) to detect macrophages, or rabbit polyclonal anti-phospho-p65 (Ser276, Santa Cruz Biotechnology; 1:100) to detect NF-κB pathway activation. Fluorescent *in situ* hybridization was performed using a probe for the conserved portion of prokaryotic 16S rRNA.

### Morphometry

To quantify neutrophils and alveolar macrophages in alveolar tissue, these cells were identified by specific immunostaining, counted in ten randomly non-overlapping tissue fields and divided by the total number of alveoli present. Airway wall remodelling was evaluated by measurement of subepithelial connective tissue volume density (VV_sub_) according to published recommendations^3^,^22^. Only cross-sectional distal airways, covered predominantly by Club cells, were analysed. Emphysematous changes of lung parenchyma were quantified using alveolar septal perimeter measurements and measurement of mean linear intercept on ten randomly chosen fields of alveolar tissue at × 200 original magnification. All morphometric measurements were made using Image-Pro Express software (Media Cybernetics, Silver Springs, MD).

### Immunodetection of IgA/NTHi binding

Fifty microlitres of a 0.2-mg ml^−1^ solution of NTHi (prepared as described above) was adsorbed onto a nitrocellulose membrane using a 96-well vacuum manifold. The membrane was washed twice with 0.1% PBS-Tween20 and then blocked for 30 min. After another two washes with 0.1% Tween20 in PBS, human SIgA from pooled colostrum (Sigma-Aldrich) was added and incubated for 1 h. The membrane was again washed twice with 0.1% Tween20 in PBS, blocked for 30 min and then incubated with rabbit polyclonal anti-IgA (Dako, Carpinteria, CA) to detect binding between NTHi and human IgA.

### NF-κB measurement

Nuclear extracts were prepared from flash-frozen lung tissue using the NE-PER kit (Pierce). Ten micrograms of nuclear protein were separated on a 10% acrylamide gel. Western blot analysis was performed with antibodies against NF-κB p65 (Santa Cruz Biotechnology; 1:1,000) or p84 (Genetex; 1:1,000) with the Odyssey infared system (LI-COR, Lincoln, NE).

### MMP-12 and NE

Whole tissue lysates were prepared from flash-frozen lung tissue using the c*O*mplete Lysis-M kit (Roche Diagnostics, Indianapolis, IN) and protein was separated on a 10% acrylamide gel. Western blot analysis was performed with Rabbit polyclonal antibodies against MMP12 (ab52897, Abcam; 1:1,000), sheep polyclonal antibodies against neutrophil elastase (61-86-20, Invitrogen, Camarille, CA; 1:1,000) or rabbit polyclonal against β-actin (A2066, Sigma-Aldrich; 1:2,000).

### Fluorescence imaging

Fluorescence imaging was performed according to a previously established protocol^30^ Folate-PEG-Cy5 was purchased from Nanocs Inc., NY (excitation wavelength=650 nm, emission=670 nm). Animals were injected intravenously with 500 nmol kg^−1^ and fluorescent imaging was performed using a Pearl Impulse system (LI-COR). Data were collected and analysed using Pearl Impulse software (LI-COR).

### 16S rRNA quantification

Total prokaryotic burden was quantified using the Femto Bacterial DNA Quantification Kit (Zymo Research, Irvine, CA). Proprietary probes targeted the V1/V2 region of prokaryotic 16S rRNA. DNase/RNase-free water was used as a negative control.

### Microbial community analysis

Lung tissue from two WT and three pIgR^−/−^ mice was immediately flash frozen in liquid nitrogen after harvest. Twenty-five milligrams of tissue from each mouse was homogenized and total genomic DNA extracted using the DNeasy Blood and Tissue Kit (Qiagen, Hilden, Germany). DNA was quantified and normalized to 2 ng μl^−1^ (Qubit 2.0 Fluorometer) before PCR amplification. Each sample was amplified in triplicate, parallel reactions with the universal 16S rRNA gene primers 27 F (5′-AGAGTTTGATCMTGGCTCAG-3′) and 338 R (5′-GCTGCCTCCCGTAGGAGT-3′) with barcoded adaptor sequences using NEBNext Master Mix (New England Biolabs, Ipswitch, MA). The amplicons were purified using Agencourt Ampure magnetic beads (Beckman-Coulter, Brea, CA) before being pooled. Sequencing was performed with paired end 250 bp reads on an Illumina MiSeq at the Georgia Genomics Facility. The software package QIIME^53^ was used for analysis of the microbial community data set. Sequence data are available at the Dryad Data Repository: doi:10.5061/dryad.17m17.

### Chemokine measurements

KC levels were measured using Milliplex magnetic beads according to the manufacturer's instructions, with assistance from the Hormone Assay and Analytical Services Core of Vanderbilt University.

### Statistical analysis

Mice were randomly assigned to the study groups and, where possible, researchers were blinded to the study groups until the time of statistical analysis. All animals were included in each analysis. Results are presented as mean±s.d. unless otherwise indicated. For experiments conducted over several time points or with multiple comparisons, a two-way analysis of variance with a Bonferroni post test was used. Pair-wise comparisons were made using *t*-tests. *P*<0.05 was considered to be significant.

## Additional information

**How to cite this article:** Richmond, B. W. *et al.* Airway bacteria drive a progressive COPD-like phenotype in mice with polymeric immunoglobulin receptor deficiency. *Nat. Commun.* 7:11240 doi: 10.1038/ncomms11240 (2016).

## Supplementary Material

Supplementary Information Supplementary Figures 1-8
