# Supplementary material for: Airway bacteria drive a progressive COPD-like phenotype in mice with polymeric immunoglobulin receptor deficiency
Source: Nat Commun. 2016 Apr 5;7:11240. doi: 10.1038/ncomms11240 (PMC4822073; doi:10.1038/ncomms11240)
Supplement: Supplementary Information — Supplementary Figures 1-8 [file ncomms11240-s1.pdf]

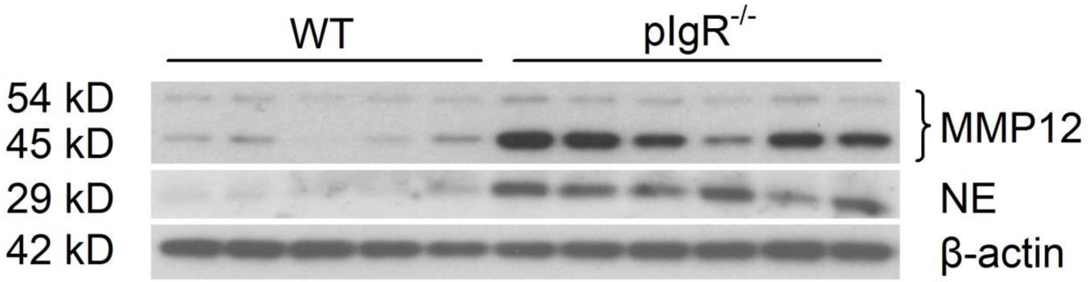

Supplementary Figure 1 Increased spontaneous MMP-12 and NE in pIgR$^{-/-}$ mice

Western blot for MMP-12 (two bands at 45 and 54 kDa) and NE (29 kDa) in lung tissue from 12 month-old WT and pIgR$^{-/-}$ mice, as shown in **Fig. 2h**.

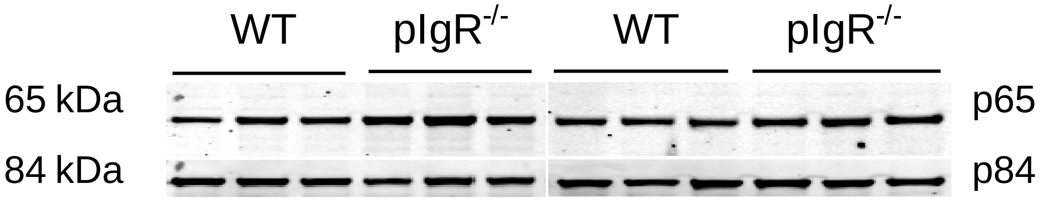

Supplementary Figure 2 Increased spontaneous NF-κB activation in pIgR$^{-/-}$ mice

Western blot showing p65 component of NF-κB in nuclear protein extracts from lungs of 12 month-old pIgR$^{-/-}$ mice and age-matched controls, as shown in **Fig. 3c**.

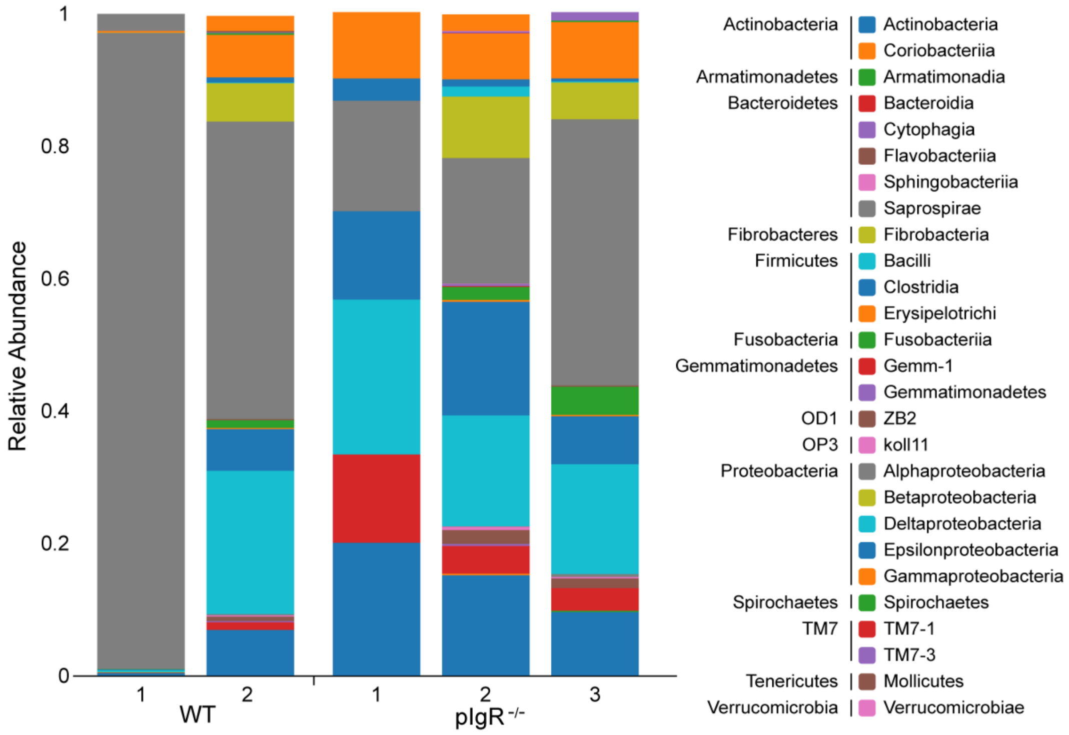

Supplementary Figure 3 Distribution of bacterial phyla and classes in lung tissue in WT and pIgR$^{-/-}$ mice

Distribution of bacterial phyla and classes in lung tissue as determined by 16S sequencing from lungs of individual 12 month-old WT and pIgR$^{-/-}$ mice.

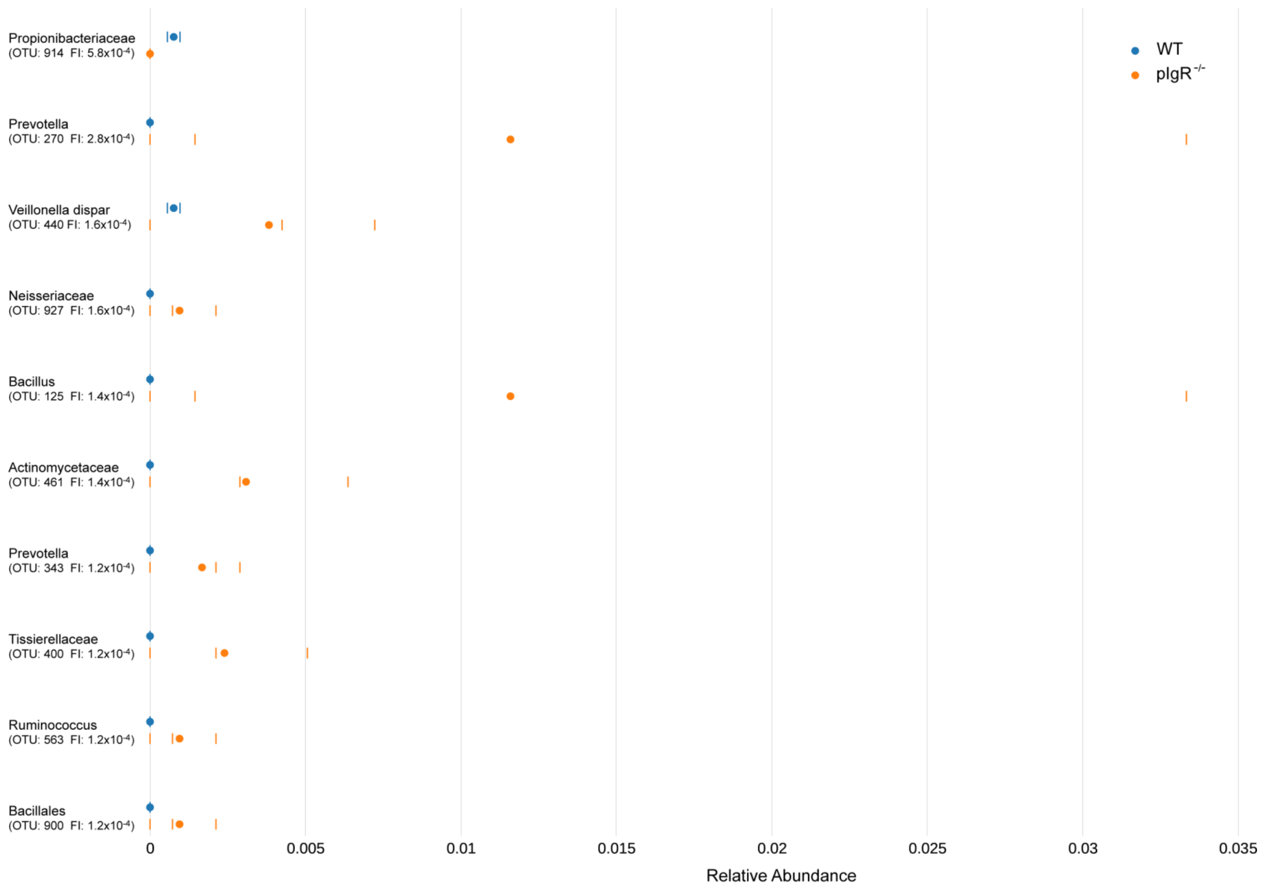

Supplementary Figure 4 Average abundance of OTUs with feature importance by genotype

Average abundance of OTUs (circle) as ranked according to random forest analysis of important features (FI) in treatment analysis of all sequences (error rate = 0.75% ± 0.5% SD). Abundance of OTUs in individual samples are represented as the tick mark. WT mice are shown in blue and pIgR$^{-/-}$ mice are shown in orange.

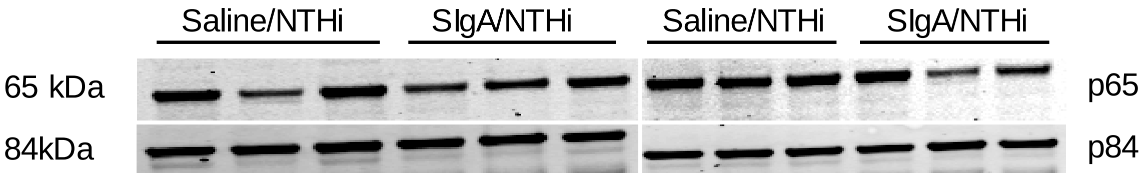

Supplementary Figure 5 Exogenous SIgA reduces NF-κB activation induced by NTHi lysates

Western blot showing p65 component of NF-κB in nuclear protein extracts from lungs of 2 month-old pIgR$^{-/-}$ mice pretreated with intratracheal SIgA or normal saline 1 hour prior to NTHi nebulization and harvested 24 hours later, as shown in **Fig. 4g**.

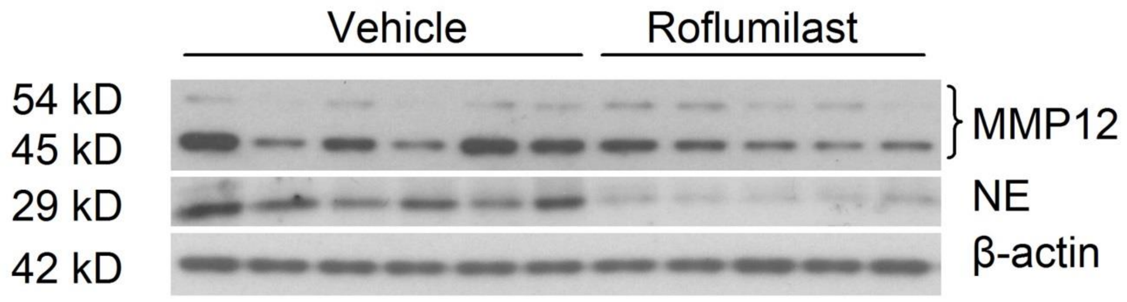

Supplementary Figure 6 Roflumilast reduces MMP-12 and NE levels in pIgR$^{-/-}$ mice

Western blot for MMP-12 (two bands at 45 and 54 kDa) and NE (29 kDa) in lung tissue from 12 month-old pIgR$^{-/-}$ mice treated with roflumilast or vehicle, as shown in **Fig. 6f**.

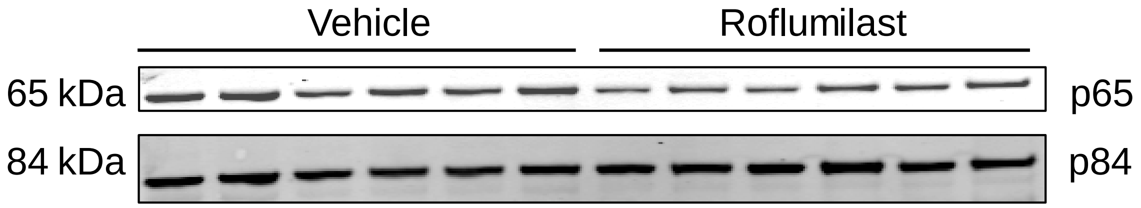

Supplementary Figure 7 Roflumilast reduces NF-κB activation in pIgR$^{-/-}$ mice

Western blot showing p65 component of NF-κB in nuclear protein extracts from lungs of 12 month-old pIgR$^{-/-}$ mice treated with roflumilast or vehicle, as shown in **Fig. 6g**.

Supplemental Figure 8 Full western blots

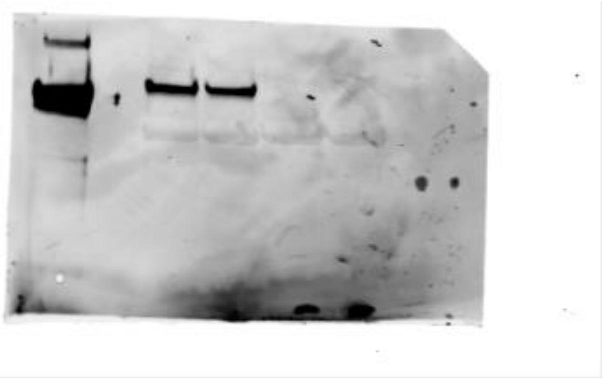

Full blot from **Fig. 1b**

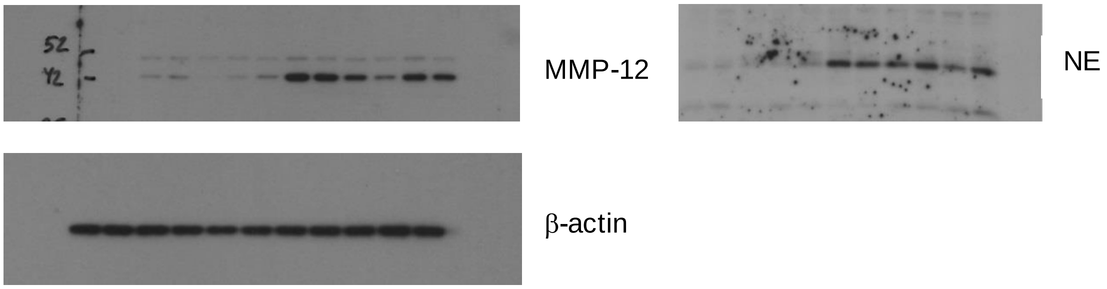

Full blots from **Fig. 2h** and **Supplementary Figure 1**

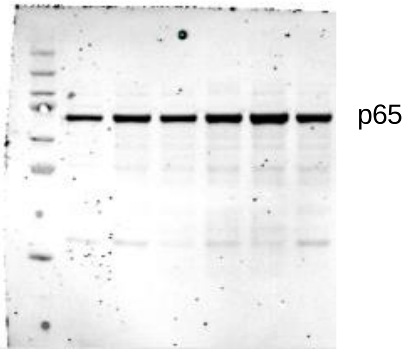

Full blot from **Fig. 3c** and **Supplementary Figure 2**, top left panel

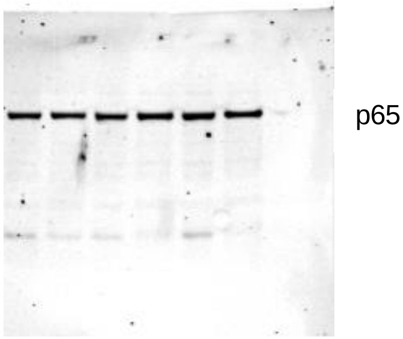

Full blot from **Fig. 3c** and **Supplementary Figure 2**, top right panel

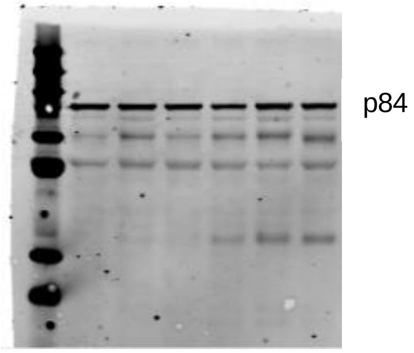

Full blot from **Fig. 3c** and **Supplementary Figure 2**, bottom left panel

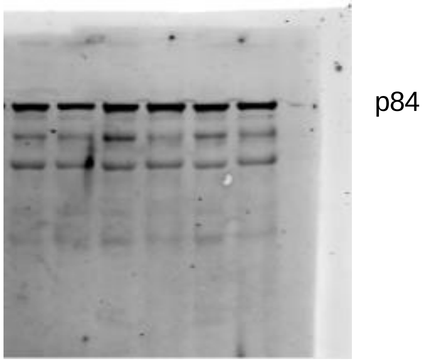

Full blot from **Fig. 3c** and **Supplementary Figure 2**, bottom right panel

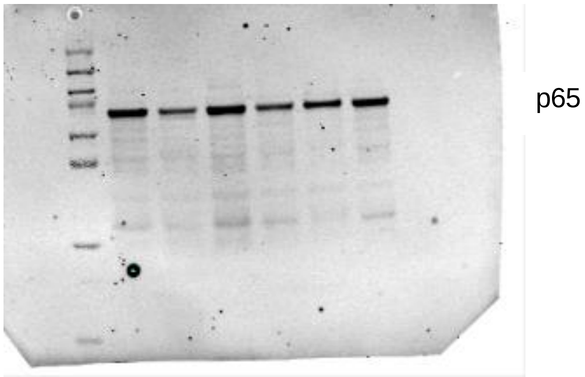

Full blot from **Fig. 4g** and **Supplementary Figure 5**, top left panel

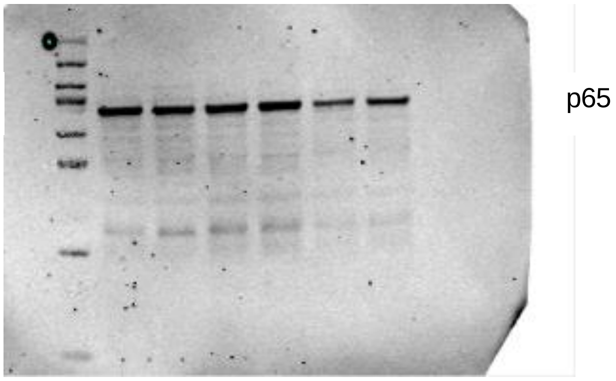

Full blot from **Fig. 4g** and **Supplementary Figure 5**, top right panel

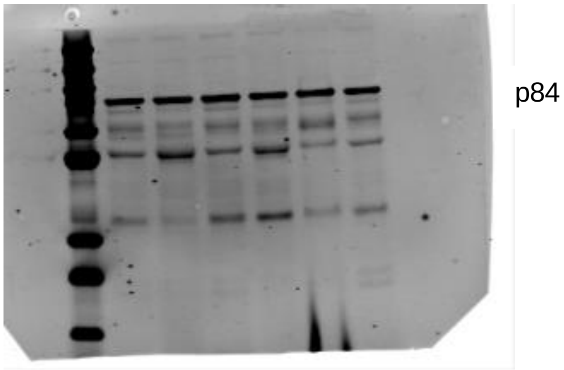

Full blot from **Fig. 4g** and **Supplementary Figure 5**, bottom left panel

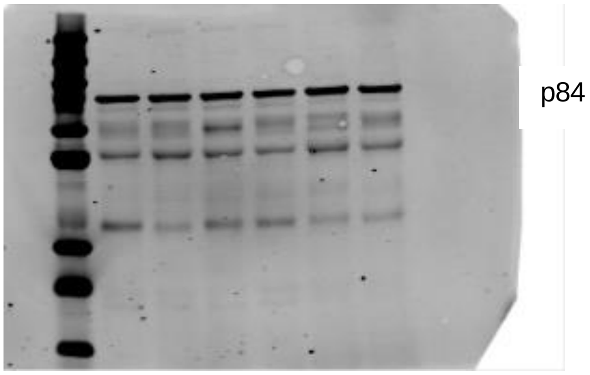

Full blot from **Fig. 4g** and **Supplementary Figure 5**, bottom right panel

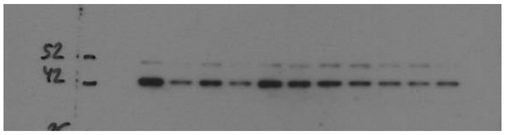

MMP-12

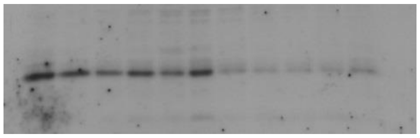

NE

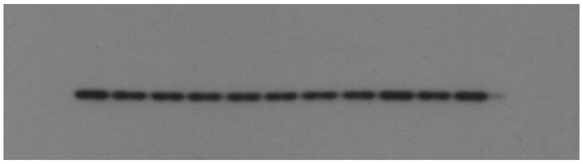

β-actin

Full blots from **Fig. 6f** and **Supplementary Figure 6**

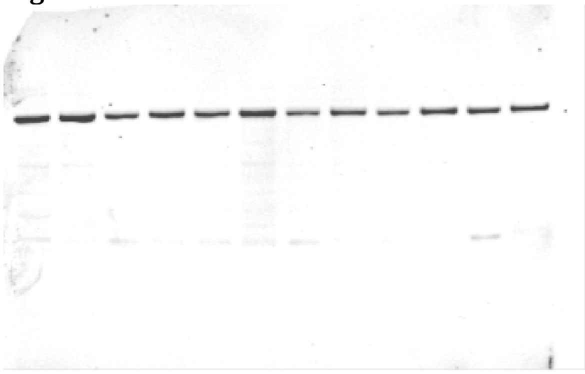

p65

Full blot from **Fig. 6g** and **Supplementary Figure 7**.

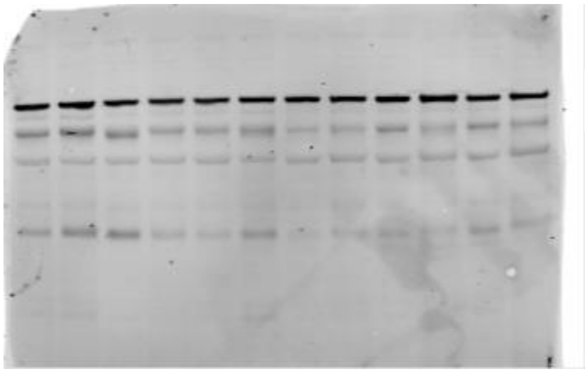

p84

Full blot from **Fig. 6g** and **Supplementary Figure 7**.
